# Numerical Simulation and Experimental Study of Cold and Hot Composite Forming of Sharp-Edged High-Strength Steel Sections

**DOI:** 10.3390/ma16216993

**Published:** 2023-10-31

**Authors:** Wenqiu Yao, Chunjing Wu, Jingtao Han

**Affiliations:** 1 Institute for Advanced Materials and Technology, University of Science and Technology Beijing, Beijing 100083, China; 2 School of Materials Science and Engineering, University of Science and Technology Beijing, Beijing 100083, China; 3 Guangzhou Sino Precision Steel Tube Industry Research Institute Co., Ltd., Guangzhou 511300, China

**Keywords:** high-strength steel, induction heating, multi-physics coupling, roll forming, cold and hot composite forming

## Abstract

This paper describes the use of cold and hot composite forming technology to produce pointed curtain wall profiles. An electromagnetic–temperature coupling model was constructed using ANSYS to study the temperature and electromagnetic field distribution during the forming process. Numerical simulation was used to optimize the process parameters to obtain the optimum heating parameters with a current of 4000 A, a frequency of 35 kHz, and a duration of 2 s. The accuracy of the model was also verified through experiments. The simulation results show that the use of a conductive magnet can improve the induction heating efficiency, increasing the heating frequency and the temperature peak; however, it also increases the temperature difference. Sharp-corner curtain wall profiles were successfully produced using the optimized process parameters. The temperature of the heating zone was measured using an infrared thermal imager, and the relative errors between the maximum heating temperature obtained from the simulation and the actual measured values were 5.37% and 5.02%, respectively, indicating that the finite element model performs well in terms of prediction.

## 1. Introduction

As the requirements for low carbon emissions and energy conservation become increasingly stringent, using high-strength steel in the construction industry can reduce its weight and improve cost effectiveness. Correspondingly, it can reduce the amount of welding and painting work, enhance the fatigue life of structures, and, by lowering steel consumption, it significantly reduces energy and resource consumption [1,2]. Due to the high yield strength and low elongation of high-strength steel at room temperature [3], defects such as twist [4], longitudinal curvature [5], flare [6], and end deformation [7] are prone to occur during the roll forming process. Phenomena like cracking at the corners [8,9] and thickness reduction [10] can also occur. Plastic deformation can also arise at the crossroads during roll forming [11,12], leading to strain hardening [13] and residual stress in the material [14,15], thereby affecting the performance of the steel sections [16,17]. The bending radius can only be increased to avoid defects in the bending zone of high-strength steel forming [18], which affects its range of use in later stages.

To address the formability of the bending zone in high-strength steel, researchers, both domestically and internationally, have adopted the method of localized induction heating. Due to the increased flexibility of high-strength steel with the rise in forming temperature [19,20], introducing induction heating can enhance the product quality and shape accuracy during the forming process. It can also overcome defects such as corner cracking and thickness reduction produced by rolls. Obaidi and Eyercioglu et al. [21,22] found that localized heating can reduce spring back and improve the accuracy of sheet bending. Yan [23] suggested using induction heating to study the warm roll forming of high-strength steel. Peng [24] found that using induction heating for forming at temperatures above 900 °C can result in localized thickening of the U-rib.

Section steel roll forming is divided into open and closed sections based on the cross-sectional shape [25]. Closed-section steel refers to a completed started section, such as a rectangular tube. Open-section steel refers to an open-constructed area, such as channel steel. Some scholars have studied closed-section sharp-edged steel. Wang [26] used the induction heating method to develop and produce high-strength steel rectangular tubes with qualified sharp edges. Mehari [27,28,29] carried out a stress–strain analysis of the roll forming process of high-strength rectangular tubes using induction heating. However, open-section sharp-edged steel has yet to be reviewed. The sharp-edged steel evaluated in this study has an open cross section and is mainly used in the curtain wall industry. In the field of curtain walling, the use of sharp-edged steel sections is crucial as they are used to support and connect the components of the curtain wall, ensuring a strong and secure connection. The use of cold and hot composite forming technology to produce sharp-edged steel profiles can better meet the requirements of curtain wall design while improving the overall quality and visual effect. It also opens up new innovative avenues for curtain wall design and construction, providing new opportunities for optimizing building appearance and structure.

The purpose of this study was to propose a cold and hot composite roll forming process to form high-strength precision profiles to be used in curtain walls instead of steel profiles. The forming method proposed in this paper can form high-strength precision profiles used in curtain walls from high-strength precision profiles with sharp openings and corners. In addition, it can meet the design requirements for achieving the decorative effect of steel profiles with rounded corners and large-area-glass-supported structures, slender components, and fire-resistant curtain walls. The design requirements require precise shape accuracy, with the external rounded corner being almost circular. Domestically and internationally, sharp-edged steel is typically manufactured using direct welding techniques with steel plates [30]. However, the welding process can introduce residual stresses, affecting overall strength [31,32]. Ultimately, comprehensive grinding is required, polluting the environment and wasting resources. This paper proposes, for the first time, the use of cold and hot composite forming technology to manufacture sharp-edged steel. First, induction heating is used to heat the pre-formed steel section at the corners locally, followed by roll forming, resulting in open-section sharp-edged steel. Taking advantage of the increased temperature from induction heating, which improves the plasticity of the material in the bending zone, high-strength steel becomes easier to form during the process. Using the cold and hot composite forming process, as opposed to the direct welding of steel plates, the generation of residual stresses can be avoided, subsequent grinding processes can be reduced, and cost can be lowered, while preventing resource wastage. Moreover, the forming process can be controlled by controlling the heating current, frequency, and duration, further enhancing the forming precision and consistency of sharp-edged steel. However, the current forming speed is 2 m/min, which is much lower than the production speed of the traditional roll forming process. Due to the high efficiency and speed of induction heating, its high energy utilization rate, and its independence from the material and thickness of the plate, it is suitable for large-scale industrial production [33]. This study is important for the development and improvement of steel curtain walls. By using cold and hot composite forming technology, it is possible to produce steel sections with sharp corners, further promoting innovation and development in the field of construction.

Using the FEM, essential parameters in the temperature field of heat transfer can be solved [34]. In induction heating technology, since traditional temperature measurement methods cannot directly obtain the temperature distribution inside the material, researchers use the finite element method to study the temperature field. Fang and Cai [35,36] established finite element models for induction heating and bending forming of round and square tubes. They discovered the influence of process parameters such as the feed rate and heating time on the forming results. Their study indicates that the influence patterns of induction heating process parameters on the inner and outer temperatures of the tube wall and their impact on forming quality are essential. Song [37] established a finite element model for electromagnetic induction heating of the crankshaft. The study employed electromagnetic–thermal coupled analysis, investigating the mutual influence between the electromagnetic field and temperature distribution during the induction heating process. By optimizing the coil structure and process parameters, researchers found that magnetic permeability is significantly influenced by the temperature and magnetic field during induction heating. This indicates that changes in temperature and magnetic field strength impact magnetic permeability during the induction heating process. Zhou, Li, and Zhang [38,39,40] applied induction heating technology to steel plate heating and established a finite element model for steel plate induction heating. Through optimizing process parameters, considering electromagnetic–thermal coupling effects, and using magnetic flux field concentrators, the efficiency and accuracy of induction heating technology have been significantly improved, bringing potential application advantages to fields such as material processing, heat treatment, and forming. Furthermore, a previous study indicated that installing magnetic flux field concentrators in the coil can effectively enhance heating efficiency, thereby expanding the application range of induction heating processes [41]. As the magnetic flux field concentrators’ width and length increase, the temperature change rate becomes faster, and the temperature distribution becomes more uniform [42].

## 2. Mathematical Model

### 2.1. Electromagnetic Field Model

The mathematical model of the electromagnetic field during the induction heating process of sharp-edged steel is based on Maxwell equations. The differential form of Maxwell equations can be represented as (1)–(4) [43].

(1) $$\nabla \times H=J+\frac{\partial D}{\partial t}$$

(2) $$\nabla \cdot E=-\frac{\partial B}{\partial t}$$

(3) ∇⋅D=q

(4) ∇⋅B=0

where the relationships between H, J, E, D, and B are as follows:

(5) D=εE

(6) B=μH

(7) J=σE

where H is the magnetic field strength, J is the current density, E is the electric field strength, B is the magnetic induction strength, D is the potential shift, q is the charge bulk density, ε is the dielectric constant, μ is the magnetic permeability, and σ is the electrical conductivity.

By introducing the vector magnetic potential A and scalar electric potential V, they, respectively, satisfy

(8) B=∇×A

(9) $$E=-\nabla V-\frac{\partial A}{\partial t}$$

Substituting (6) and (8) into (1) gives the following equation:

(10) $$\nabla \times \frac{1}{\mu }\nabla \times A=J+\frac{\partial D}{\partial t}$$

Therefore, the control equation for eddy current distribution during the induction heating process is

(11) $$\nabla \times \frac{1}{\mu }\nabla \times A=J_{s}+j\omega \gamma A$$

where $J_{s}$ represents the field current density and ω represents the field current angular frequency.

Analysis of the electric and magnetic fields requires consideration of boundary conditions. The electric field boundary condition obtained from the integral equation set at the interface is [44]

(12) $$E_{1t}=E_{2t}$$

(13) $$D_{2n}-D_{1n}=\sigma_{s}$$

where $\sigma_{s}$ represents the charge density.

To perform magnetic field analysis using the respective relative magnetic permeabilities $\mu_{r1}$ and $\mu_{r2}$ of each material, the current density on the interface is assumed to be $J_{s}$. The magnetic field condition on the interface is as follows:

(14) $$B_{1n}=B_{2n}$$

(15) $$H_{2t}-H_{1t}=J_{1}$$

where $J_{1}$ represents the current density ratio.

When the conductivity differs between dielectrics, the relationship between current densities as it passes from one dielectric to another is as follows:

(16) $$J_{1n}=J_{2n}$$

To reflect real-world conditions, an outer boundary is established at a distance from the sensor where the magnetic vector potential is set to zero.

(17) A=0

### 2.2. Temperature Field Model

The temperature field in the heating zone of sharp-edged steel follows not only the first law of thermodynamics but also obeys Fourier’s law during the heat conduction process, as shown in the following equation [44]:

(18) $$c\rho \frac{\partial T}{\partial t}+\nabla \left(-\lambda \nabla T\right)=Q_{v}=\sigma E^{2}$$

where ρ is the density, c is the specific heat capacity, T is the temperature, λ is the thermal conductivity, and $Q_{v}$ is the thermal power density.

In solving transient processes, priority should be given to considering both convection and radiation as the two heat transfer modes [45]. The boundary conditions in heat transfer calculations can be described as [44]

(19) $$k\nabla T\cdot n+h\left(T-T_{amb}\right)+\varepsilon \sigma_{sb}\left(T^{4}-T_{a}^{4}\right)=0$$

where $T_{amb}$ is the ambient temperature, n is the boundary surface normal vector, h is the surface convection heat transfer coefficient, ε is the surface emissivity, and $\sigma_{sb}$ is the Stefan–Boltzmann constant, which is generally set to $\sigma_{sb}=5.67\times 10^{-8}W/\left(m^{2}\cdot K^{4}\right)$.

### 2.3. Geometric Model

**Figure 1:**
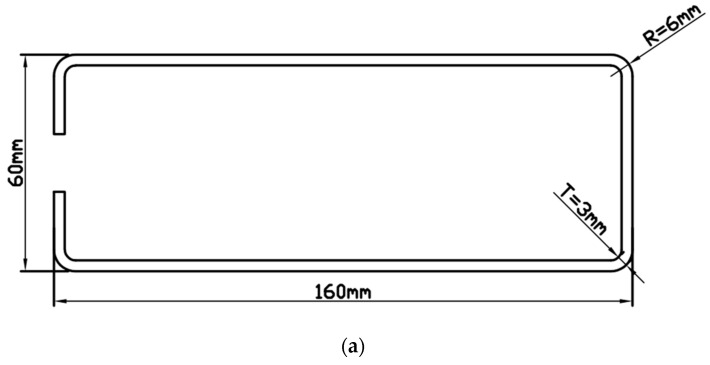

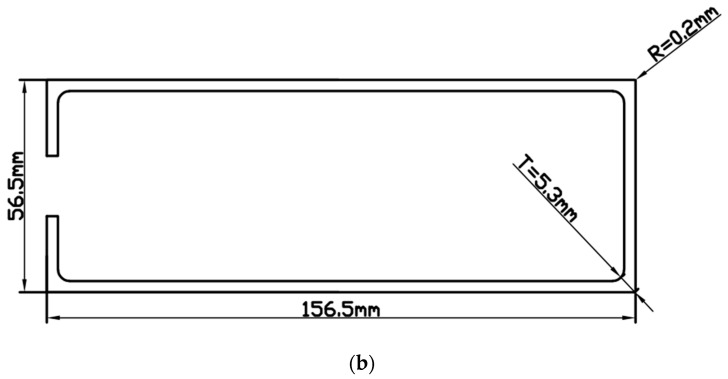
External dimensions of steel sections: (**a**) external dimensions of the preformed section; (**b**) dimensions of the profile after forming.

**Figure 2:**
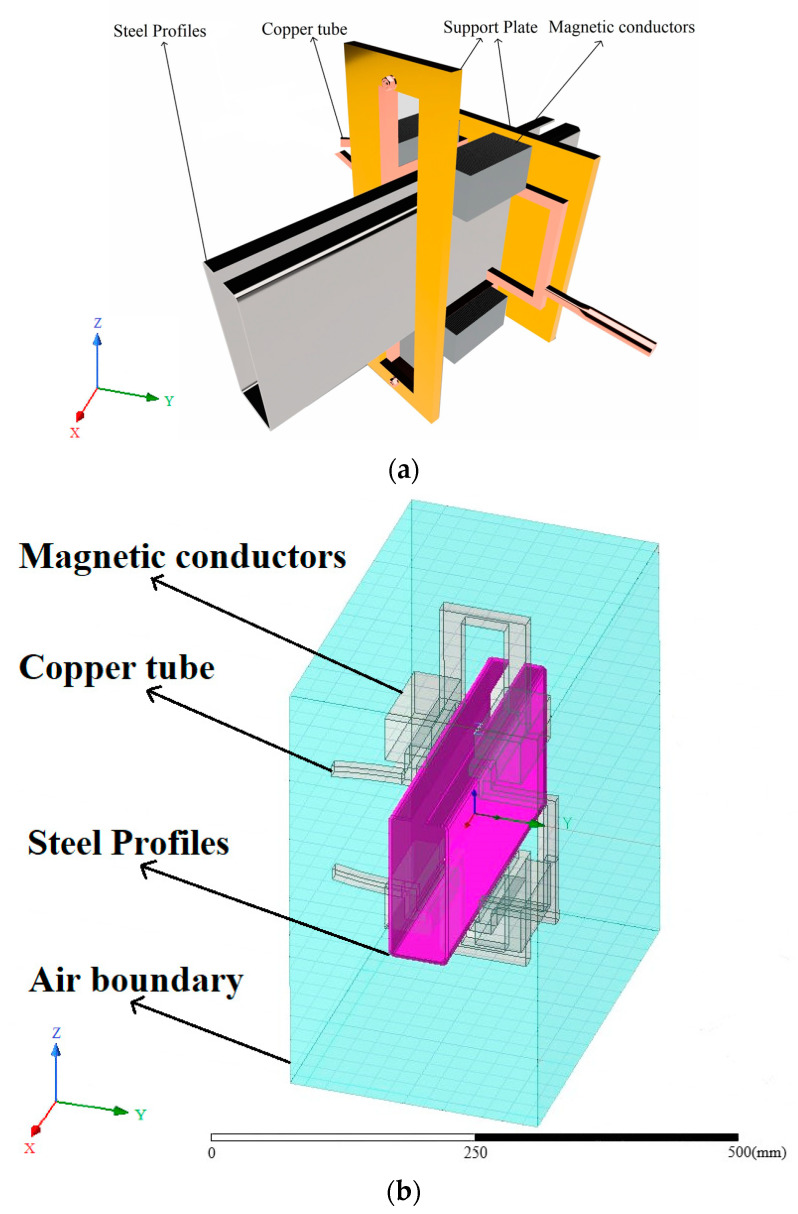
Geometric model: (**a**) three-dimensional model; (**b**) three-dimensional model and boundaries.

Figure 1 shows that the pre-formed-steel-section dimensions are as follows: length 160 mm, width 60 mm, external corner radius R = 6 mm, and corner thickness T = 3 mm. After forming, the sharp-edged-steel-section dimensions are as follows: length 156.5 mm, width 56.5 mm, outer corner radius R = 0.2 mm, and corner thickness T = 5.3 mm. Figure 2 shows a three-dimensional model of local induction heating of the sharp-edged steel using an induction heating coil for heating. The induction heating coil includes a copper tube, magnetic flux field concentrators, and support plates. Specifically, the pre-formed steel section passes through the induction coil, and the magnetic flux field concentrators are installed on the outside of the induction coil copper tube. Support plates are installed at both ends of the copper tube to provide support. This design structure aims to achieve effective heating of the corner of the sharp-edged steel.

## 3. Numerical Simulation

The numerical simulation consists of three modules: pre-processing, solving, and post-processing.

### 3.1. Pre-Processing

The first step involved using drawing software to create a three-dimensional model and saving it in a universal graphic file format. Next, this model was imported into ANSYS to generate the geometric model and perform necessary optimizations and simplifications. During this process, we did not consider the vortex loss that the internal cooling water might cause, nor did we feel the motion properties of the sharp-edged steel during the heating process. Finally, the generated geometric model was input into the Maxwell module for subsequent simulation and analysis.

**Figure 3:**
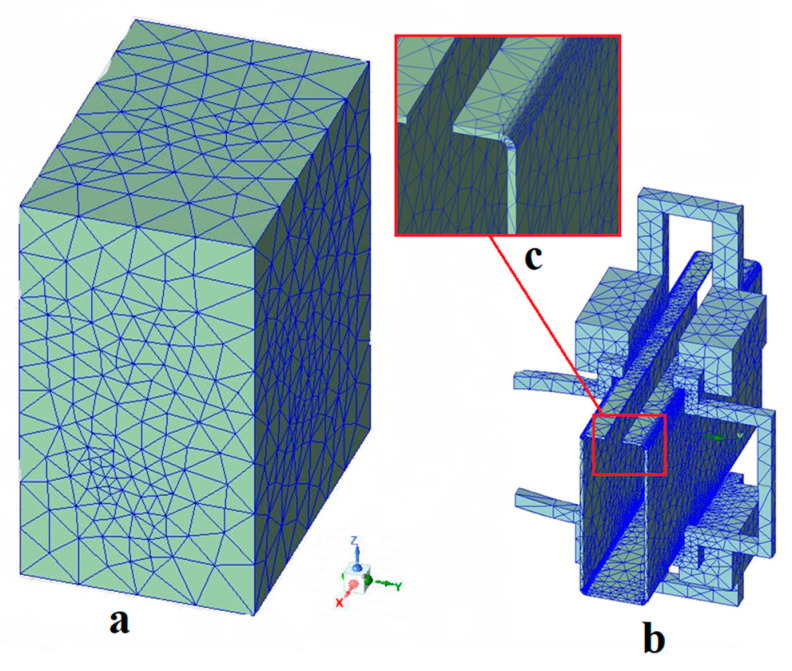
Three-dimensional model mesh partition diagram: (**a**) the meshing of the 3D model with air boundaries; (**b**) the meshing of the 3D model including the copper pipe and the magnetic flux field concentrators; (**c**) the enlarged corners of the meshing of the sharp-edged steel section.

The second step involved meshing. To simplify the computation, we assumed that the moving part of the sharp-edged steel remained stationary, and areas that did not require heating or had a low temperature were ignored. However, local mesh refinement was carried out in the corner heating zone. We used the adaptive meshing method built into the Maxwell module and chose hexahedral elements to represent the steel section. At the same time, the modeling of the induction coil, silicon steel sheet, and air used hexahedral ingredients. A schematic of this process is shown in Figure 3. Figure 3a is a three-dimensional model meshing diagram that includes the air boundary, Figure 3b is a meshing diagram that consists of the copper tube and magnetic flux field concentrators, and Figure 3c is an enlarged meshing diagram of the corner of the sharp-edged steel.

The third step involved assigning material properties to geometric entities and defining the physical domains. The relative magnetic permeability $\mu_{r}$ of the copper tube, air domain, and magnetic flux field concentrators was set to 1, 1, and 1000, respectively. Since these three did not participate in the temperature field calculation, there was no need to define additional material parameters related to the temperature field. However, for the sharp-edged steel that would participate in the coupling calculation of the electromagnetic field and temperature field, we needed to define material parameters, such as relative magnetic permeability $\mu_{r}$, resistivity ρ, thermal conductivity k, and specific heat capacity c, which vary with temperature [46].

### 3.2. Solving

Induction heating technology is a method of electrical heating that relies on two physical principles: electromagnetic induction and the Joule heating effect [47]. Therefore, when performing numerical simulations of induction heating, conducting a coupled analysis of the electromagnetic field and temperature field [48] is necessary. In this study, we employed an iterative sequential coupling solution method [49] to address the interaction between the electromagnetic and temperature fields. This approach allows for relatively accurate simulation and analysis of electromagnetic and thermal conduction phenomena during the induction heating process, providing effective numerical simulation results for optimization and design in practical applications.

### 3.3. Post-Processing

When the center temperature of the cross section is 850 °C after corner heating, with the electrical resistivity as $\rho_{t}=12\times 10^{-7}\Omega \cdot m$, relative magnetic permeability as $\mu_{r}=1$, and vacuum permeability as $\mu_{0}=12.56\times 10^{-7}\left(H/m\right)$, calculated from Equation (21), the skin depth is δ=3 mm and the required frequency is f= 33.89 kHz, as per the following formula:

(20) $$\delta =\sqrt{\frac{\rho_{t}}{\pi f\mu }}$$

We obtained the following formula:

(21) $$f=\frac{\rho_{t}}{\pi \delta^{2}\mu_{\rho }\mu_{0}}$$

In the numerical simulation study of induction heating of angle steel, the induction heating frequency, current, and heating time are the three main process parameters [39]. Given that the wall thickness of the angle steel is 3 mm and the center temperature of the heated section at the corner is 850 °C, the selected range for the induction heating frequency was from 15 kHz to 40 kHz, and the heating current range chosen was from 2000 A to 5000 A.

## 4. Results and Discussion

### 4.1. Magnetic Core Influence on Field Strength

**Figure 4:**
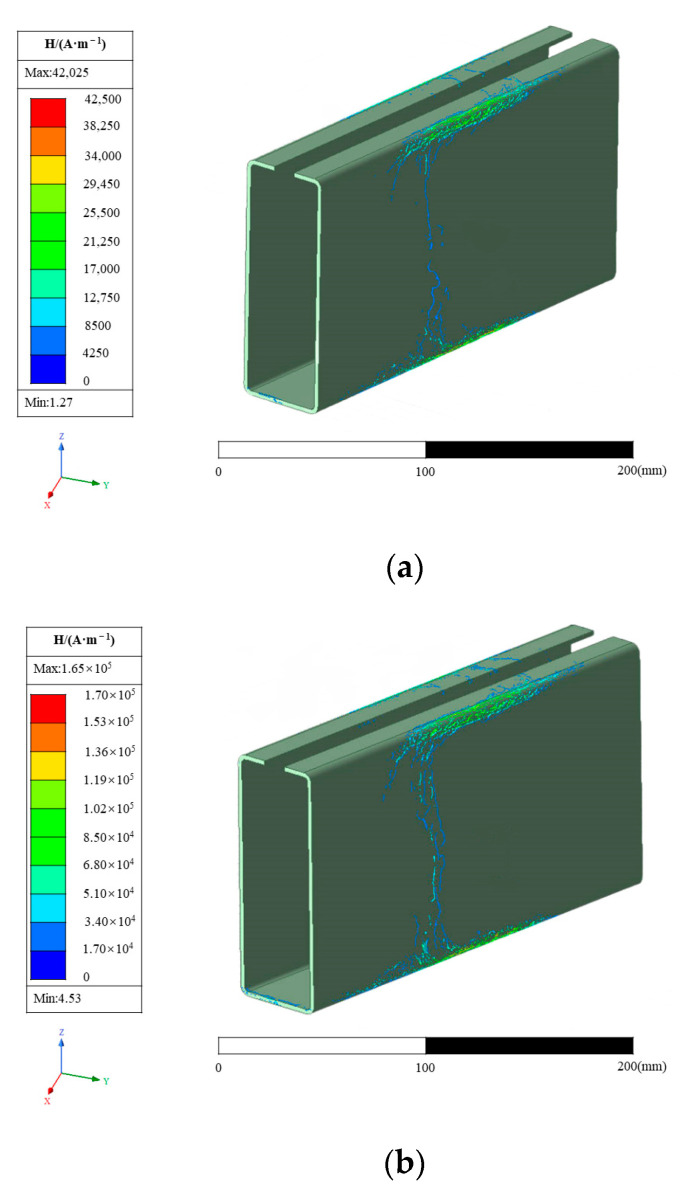
Influence of the magnetic core on magnetic field intensity: (**a**) the resulting diagram of the model without a magnetic core; (**b**) the resulting diagram of the model with a magnetic core.

The induction heating frequency was 40 kHz, and the heating current was 3000 A. The magnetic field intensity distribution with a heating time of 2 s is shown in Figure 4, where Figure 4a is the result diagram of the model without a magnetic core and Figure 4b is the result diagram of the model with a magnetic core. The relative permeability of the magnetic conductor is much higher than that of air, copper pipe, and steel, which greatly reduces the magnetic resistance of the area where it is located. Furthermore, it can “attract” the magnetic induction lines originally freely spread in the air domain to the body, so that the magnetic induction lines can be passed through the body, greatly increasing the magnetic flux in the body. By controlling the opening position, it can guide the high-density magnetic induction lines in the body to the heating area to be heated. Then, by controlling the opening position, the high-density magnetic lines in the body are directed to the heating area. Using the same simulation parameters, the ability of the conductor to control the spatial distribution of the magnetic lines and the significant increase in flux in the local area can be clearly seen.

As is shown in Figure 4a, during the induction heating process, when the sharp-edged steel is close to the induction coil, the magnetic field inside the induction coil will induce a current within the steel section. According to Lenz’s law, these induced currents will generate a magnetic field inside the conductor, which interacts with the magnetic field of the induction coil. On the surface of the steel section, due to the distribution of the current within the conductor, areas with a higher current density typically form. The path of the induced current within the steel section aims to minimize the magnetic reluctance between the induction coil and the steel section, thereby concentrating the current more on the surface of the steel [50].

As is shown in Figure 4b, when an L-shaped magnetic flux field concentrator is added around the induction coil, as current flows through the induction coil, the concentrator directs the magnetic field to the slot end, resulting in a higher magnetic field strength near the slot end. At this time, the current will also be more concentrated near the slot end [42]. The presence of the magnetic flux field concentrator can enhance the efficiency of the induction coil by increasing the current density within the coil. It can improve the efficiency of the induction heating system, achieving faster heating speeds and higher energy utilization rates [51].

As is shown in Figure 4a, near the outer surface where the sharp-edged steel is adjacent to the copper tube, the magnetic field strength reaches its maximum value of 42,025 A/m. The magnetic field strength gradually decreases along the outer surface of the sharp-edged steel. In Figure 4b, we can observe a similar trend, where the maximum magnetic field strength appears in a similar position at 165,600 A/m, which is 3.9 times higher than the total value in Figure 4a. This indicates that a substantial magnetic field area is formed between the opening position of the magnetic flux field concentrators and the corner of the sharp-edged steel. In contrast, the magnetic field strength rapidly decreases in other parts of the steel section, approaching zero [52,53,54].

### 4.2. Effect of Heating Current and Frequency on Temperature Distribution

**Figure 5:**
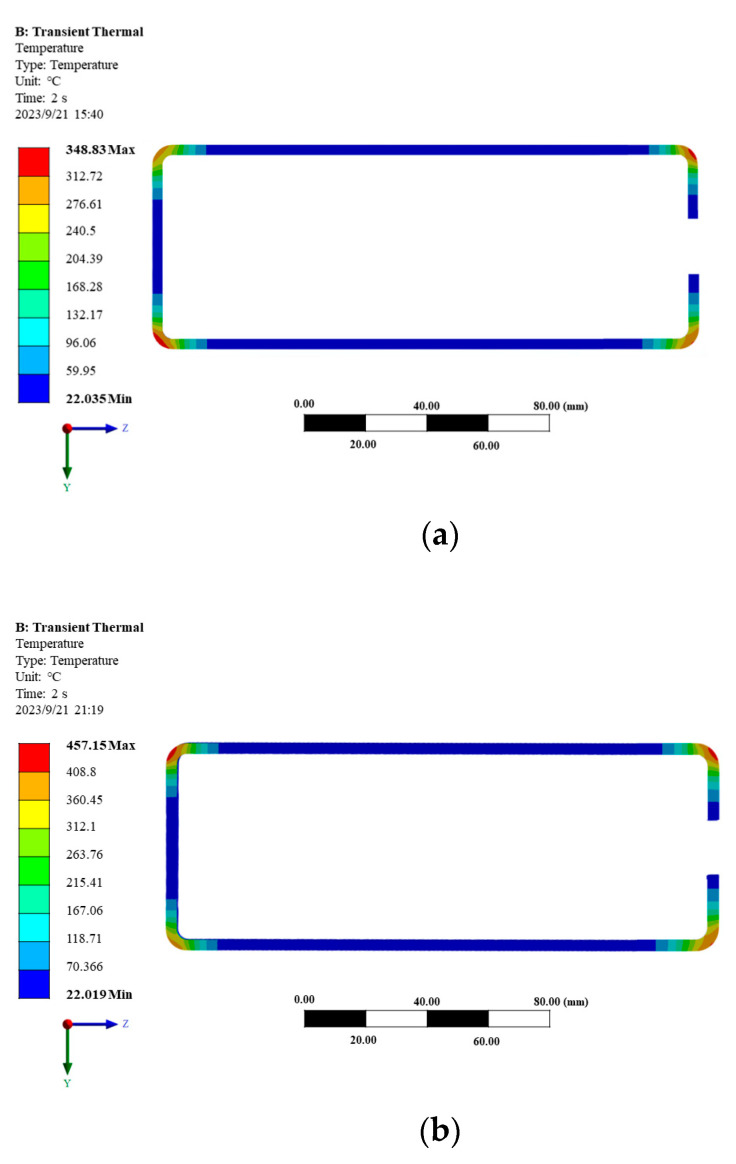

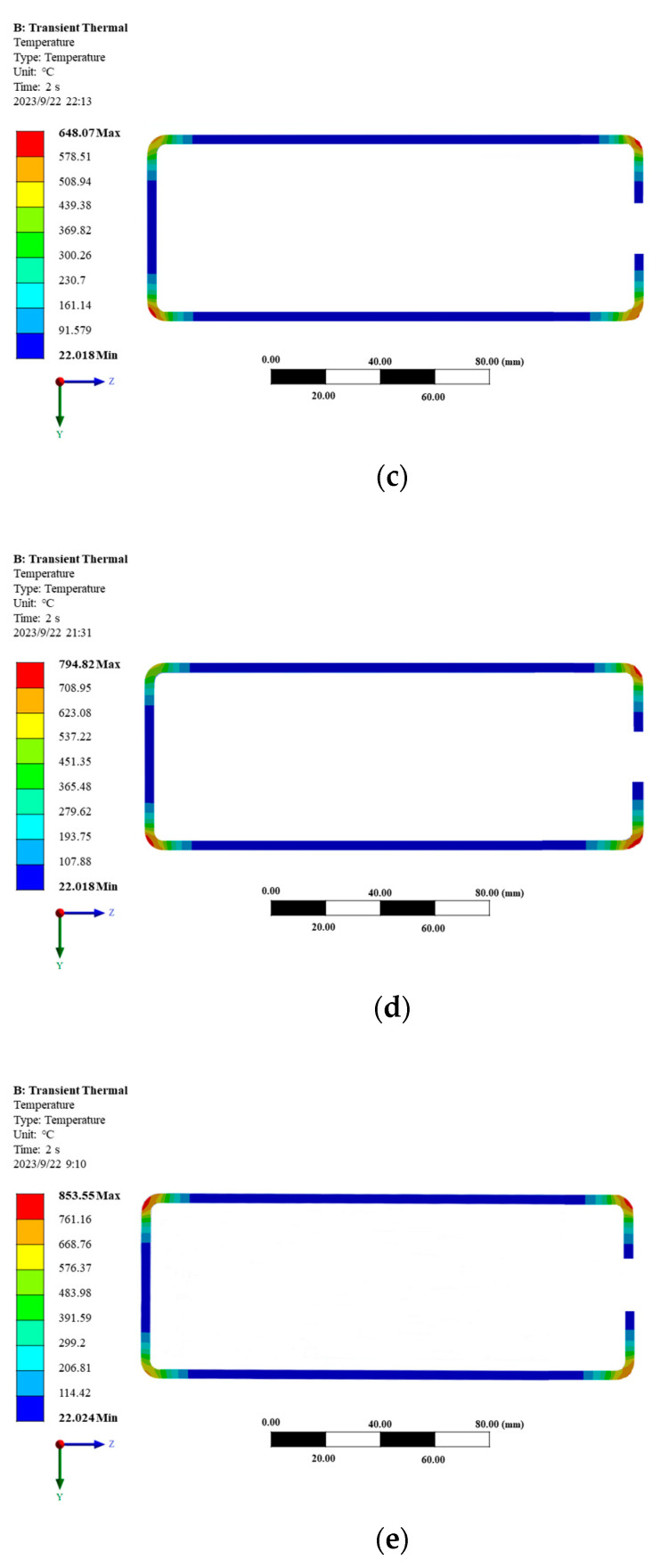

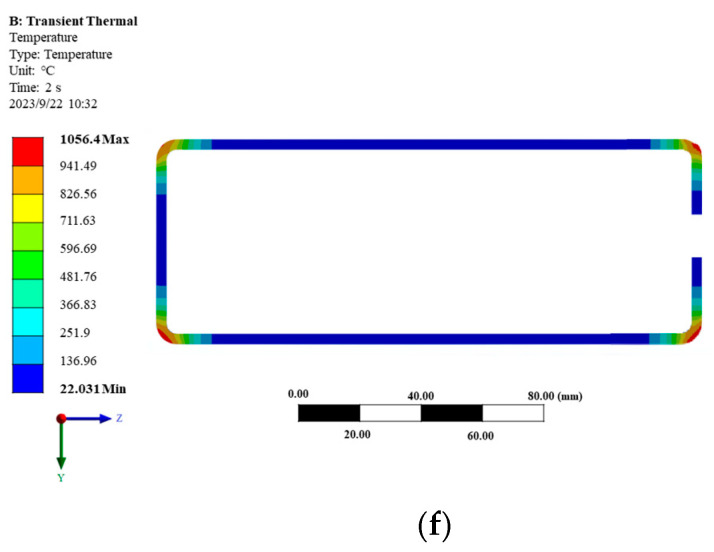
Temperature distribution diagram of the corner of sharp-edged steel at different heating frequencies. (**a**) Temperature distribution diagram with a current of 3000 A and a frequency of 15 kHz; (**b**) temperature distribution diagram with a current of 3000 A and a frequency of 20 kHz; (**c**) temperature distribution diagram with a current of 3000 A and a frequency of 25 kHz; (**d**) temperature distribution diagram with a current of 3000 A and a frequency of 30 kHz; (**e**) temperature distribution diagram with a current of 3000 A and a frequency of 35 kHz; (**f**) temperature distribution diagram with a current of 3000 A and a frequency of 40 kHz.

The model adopts a structure that includes magnetic flux field concentrators to study the effects of heating current and frequency on simulation results. The heating time was set to 2 s, the heating current was chosen as 4000 A, and the frequencies were selected as 15 kHz, 20 kHz, 25 kHz, 30 kHz, 35 kHz, and 40 kHz for individual modeling and analysis. The temperature distribution of the sharp-edged steel section is shown in Figure 5. As is shown in Figure 5, at 15 kHz, the highest temperature at the outer corner was 348.83 °C, while at 40 kHz, the highest temperature at the outer corner reached 1056.4 °C. The results indicate that when other parameters remain constant, the higher the heating frequency, the faster the temperature reaches its peak value [55].

**Figure 6:**
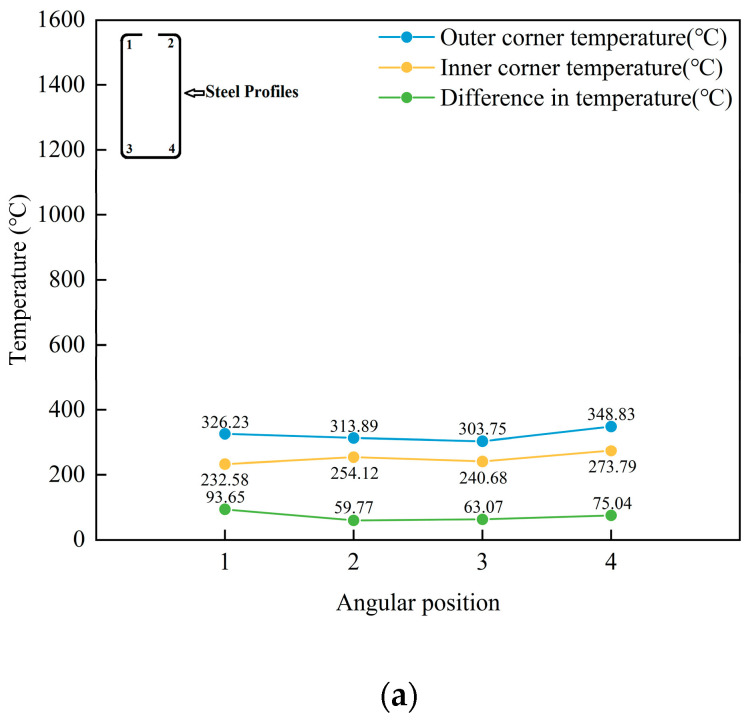

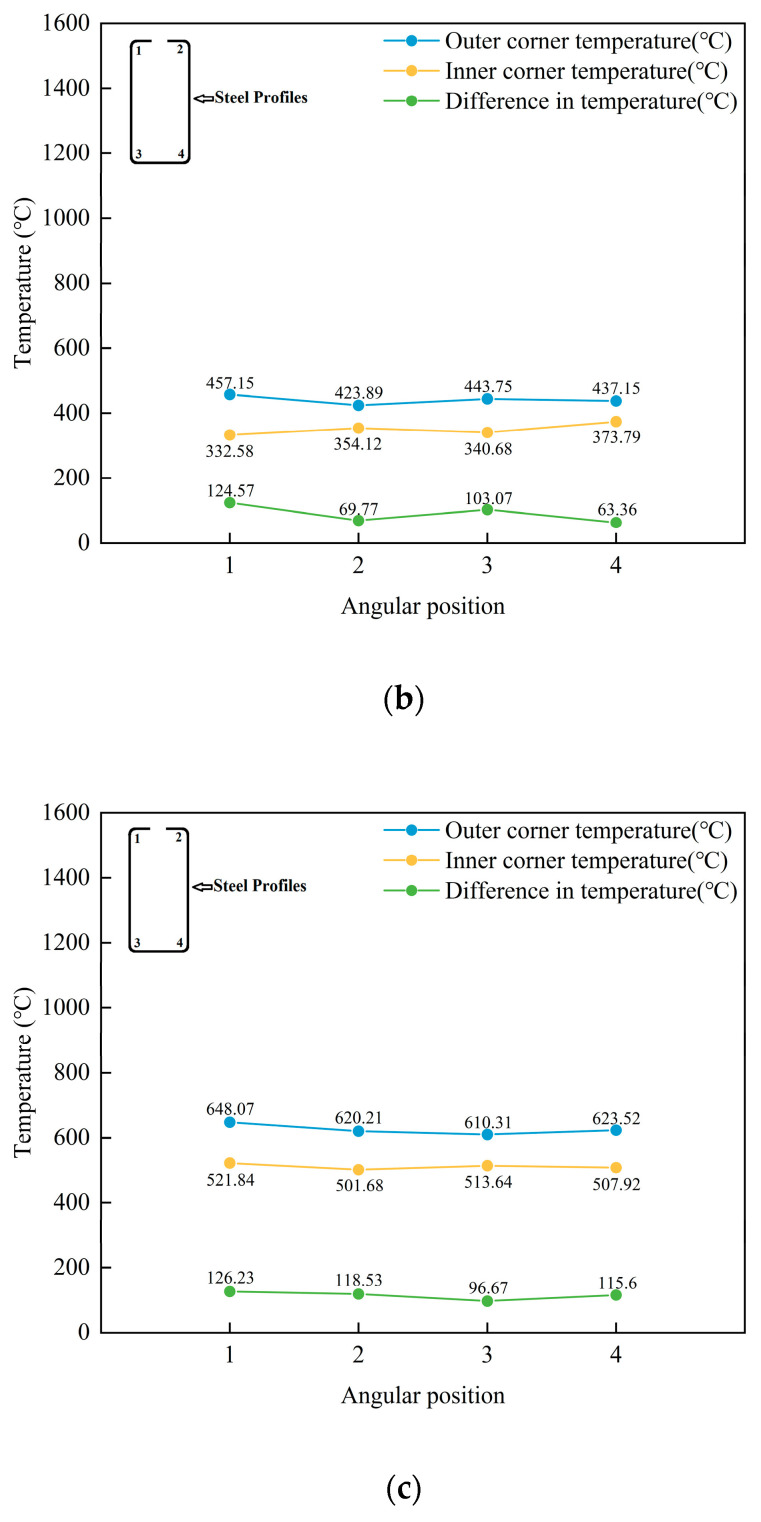

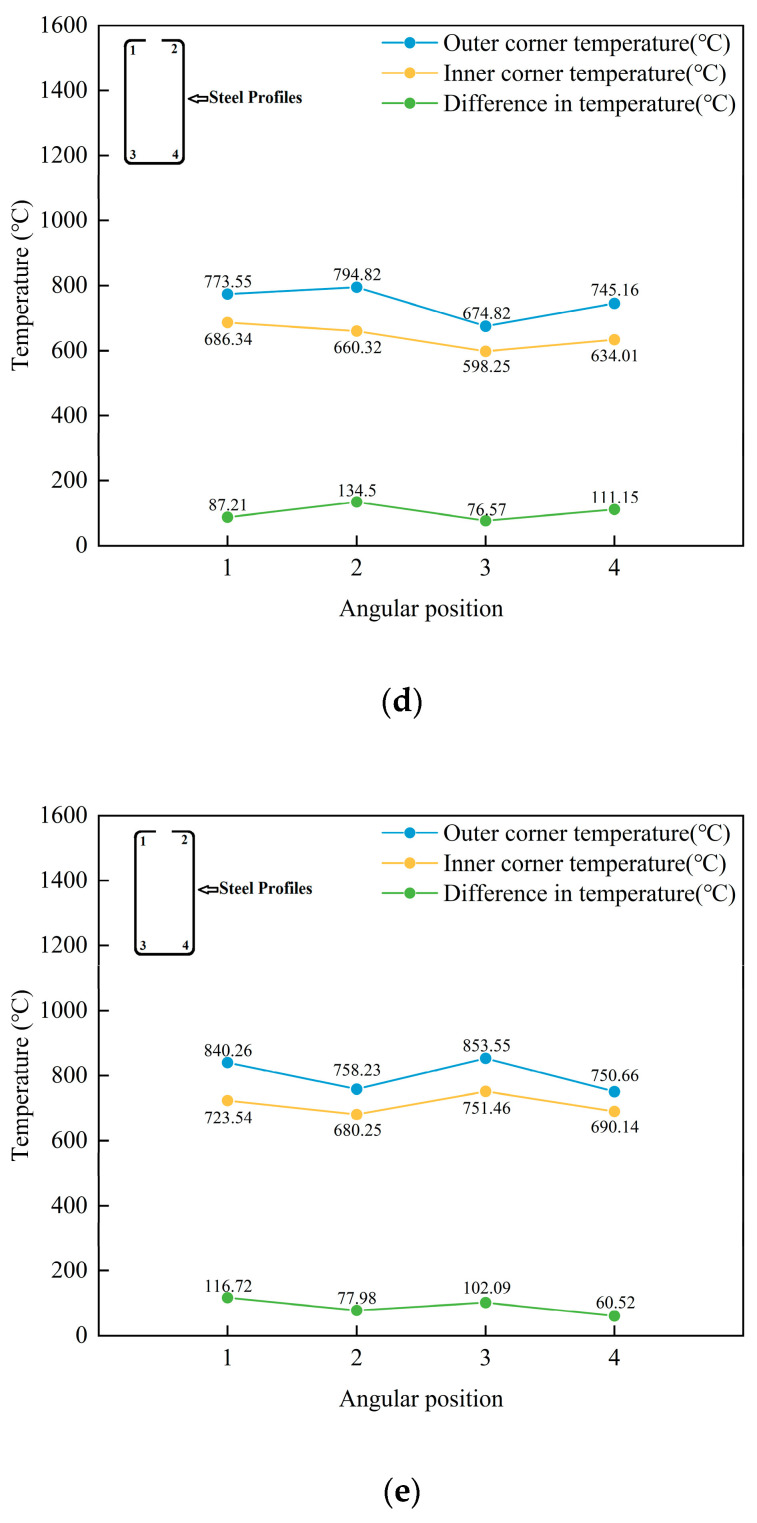

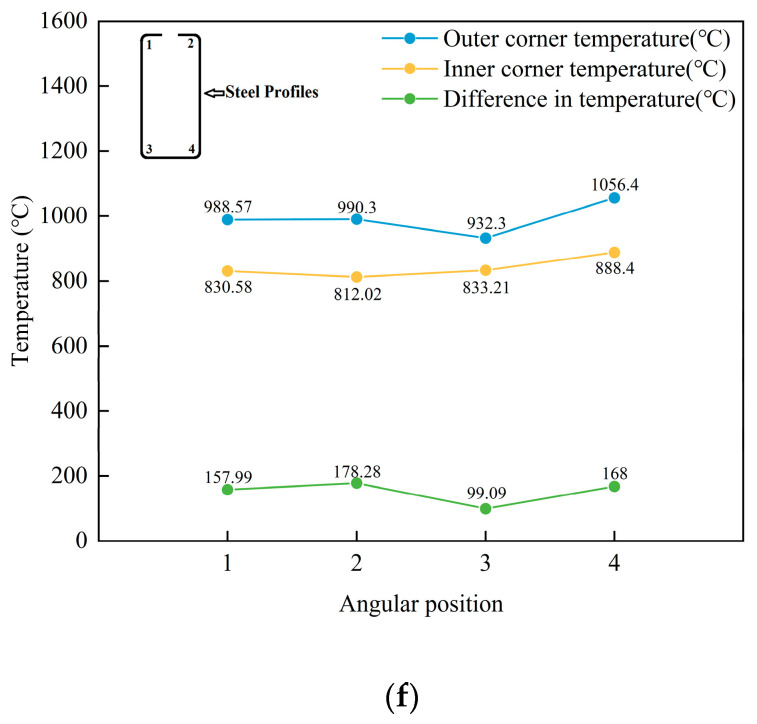
Temperature curves of the outer corner, inner corner, and temperature difference after heating at different frequencies. (**a**) Temperature curve of the corner at a current of 3000 A and a frequency of 15 kHz; (**b**) temperature curve of the corner at a current of 3000 A and a frequency of 20 kHz; (**c**) temperature curve of the corner at a current of 3000 A and a frequency of 25 kHz; (**d**) temperature curve of the corner at a current of 3000 A and a frequency of 30 kHz; (**e**) temperature curve of the corner at a current of 3000 A and a frequency of 35 kHz; (**f**) temperature curve of the corner at a current of 3000 A and a frequency of 40 kHz.

Figure 6 displays the temperature curves of the outer corner and inner corner and the temperature difference in the corner after heating with an input current of 3000 A and different current frequencies of 15 kHz, 20 kHz, 25 kHz, 30 kHz, 35 kHz, and 40 kHz, with a heating time of 2 s.

The results shown in Figure 6 indicate that during induction heating, as the heating frequency increases, eddy currents are generated at shallower positions from the surface, leading to a shift in the peak magnetic induction intensity towards the outer corner surface [56]. In a high-frequency alternating magnetic field, the current flows more concentratedly through the conductor’s surface, while the current power in the middle of the material gradually decreases [57]. With the increase in frequency, the generation of eddy currents is affected by the internal resistance of the material, causing the current to be mainly concentrated on the surface, and the current generated in the middle of the material is minor. The skin effect is a significant factor causing the peak magnetic induction intensity to shift towards the outer corner surface. During induction heating, the skin effect [58] means that a high-frequency alternating magnetic field will cause eddy currents on the material’s texture, generating heat. However, when the surface temperature of the material rises above its Curie point, the magnetism of the material changes, transitioning from its original magnetic state to a non-magnetic or weakly magnetic state. This phenomenon affects the efficiency and depth of induction heating.

**Figure 7:**
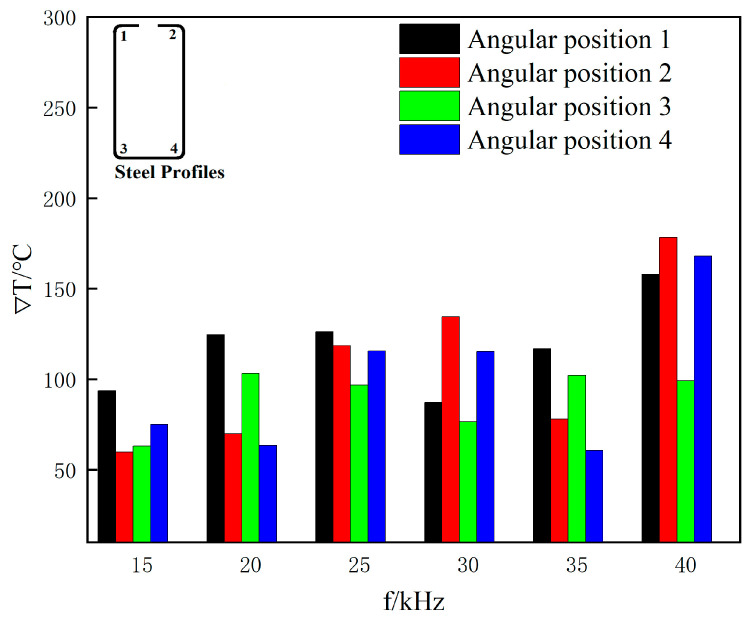
Variation in corner temperature difference with heating frequency.

The raw material for the sharp-edged steel in this study was Q355B steel produced by Anshan Iron and Steel Group Co., Ltd. in Anshan City, China. The highest temperature at which austenite and ferrite coexist in equilibrium is approximately 850 °C, calculated using the JMatPro v4.1 material property simulation software. Therefore, the center temperature of the section after heating the corner was set to 850 °C. To even out the temperature in the corners, the temperature difference (∇T) between the outer and inner corners at different heating frequencies was statistically analyzed to meet the heating requirements of uniform heat conduction in the thickness direction. The results are shown in Figure 7.

As is shown in Figure 7, as the heating frequency increased, the temperature difference continued to grow. When it increased from 15 kHz to 40 kHz with a heating duration of 2 s, the temperature difference rose by 68.7%, 198%, 57.1%, and 123.8%. When the frequency was f = 40 kHz, and the current was I = 3000 A, the simulated results measured the center temperature of the section after heating the corner to 850 °C. At this time, the temperature differences between the outer and inner corners were 157.99 °C, 178.28 °C, 99.09 °C, and 168 °C. As the heating frequency increases with a heating duration of 2 s, the temperature difference significantly rises, and the center temperature of the corner section will also increase accordingly. In conclusion, the optimal heating frequency is 40 kHz, and for ease of calculation, the corresponding movement speed of the steel section was set to 2 m/min.

Changing the frequency affects the temperature distribution of the profile. If the frequency is too high, the induced current is mainly concentrated in the surface layer of the steel profile. When the profile is heated to the Curie temperature, the surface temperature will rise sharply in the magnetic stage, mainly because of the skin effect. When the surface temperature of the steel profile exceeds the Curie temperature during the transition to the non-magnetic stage, the region under the surface layer is still maintained in the magnetic stage for the transition heating stage. At this time, the distribution of energy density along the thickness direction reaches a maximum at the surface and decreases with increasing depth. The rate of temperature increase in the outer corners is higher than in the inner corners, which are heated by conduction, due to the proximity effect [59].

### 4.3. Effect of Frequency on the Magnetic Induction Strength

**Figure 8:**
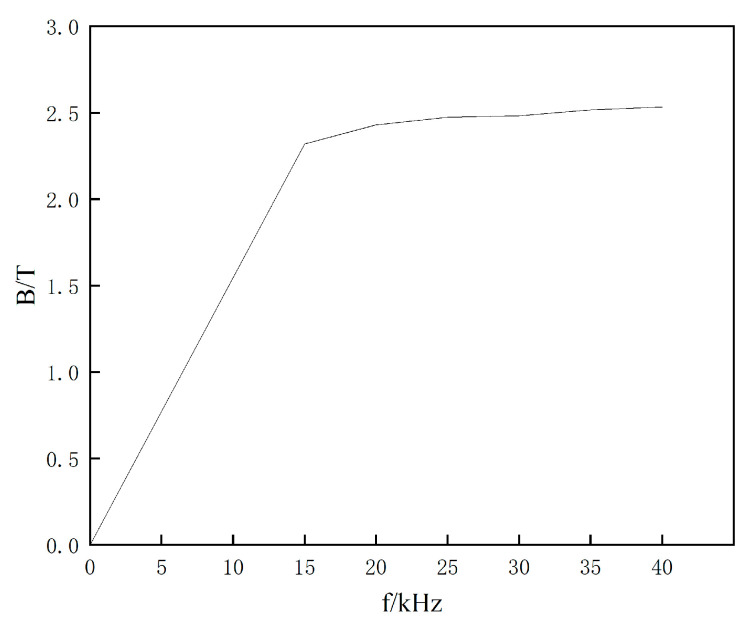
Relationship between magnetic induction intensity and different current frequencies.

Based on the data from Figure 8, when the frequency is f = 15 kHz, the peak magnetic induction intensity on the surface of the sharp-edged steel is 2.32 T. When the frequency is increased to f = 40 kHz, the peak magnetic induction intensity on the surface of the sharp-edged steel increases to 2.533 T. In comparison, it is found that when the frequency is raised from f = 15 kHz to f = 40 kHz, the magnetic induction intensity on the surface of the sharp-edged steel increases by 9.1%. This results in a 2.6-fold increase in the surface temperature of the sharp-edged steel. The results show that as the heating frequency increases, the magnetic induction intensity significantly increases, especially on the outer corner surface, thereby increasing the heating rate and temperature. However, this also leads to a larger temperature difference between the outer and inner corners, as the peak magnetic induction intensity is directly proportional to the heating frequency [37].

## 5. Experimental Validation

### Heating Temperature

**Figure 9:**
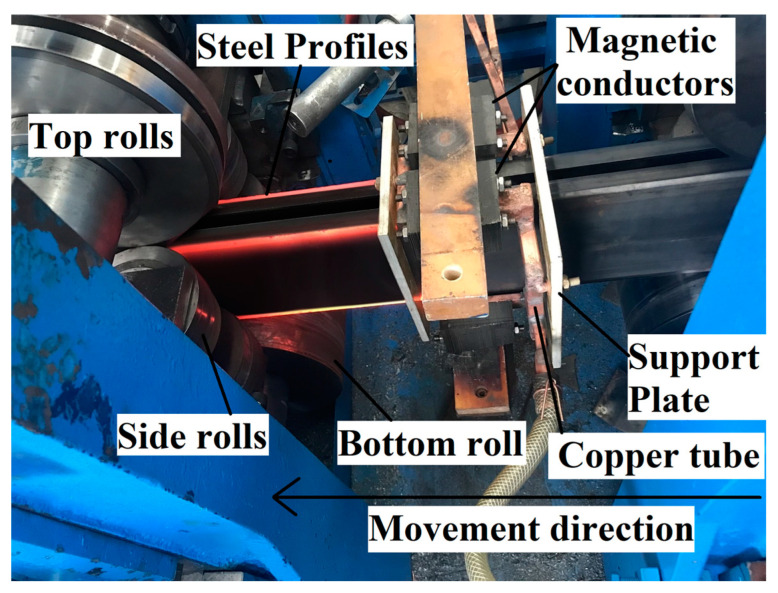
Experimental equipment.

**Figure 10:**
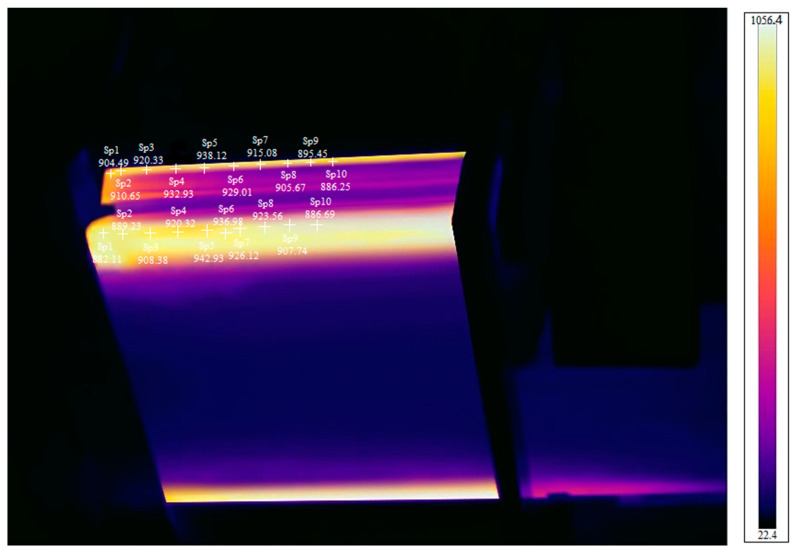
Thermal imaging picture. + in the diagram is the temperature measurement position marker.

To verify the accuracy of the results of the numerical simulation, an induction heating coil identical to the geometric model shown in Figure 2 was fabricated. The exact size specifications from the finite element model were adopted to conduct experimental studies on sharp-edged steel’s local induction during hot forming. Specifically, magnetic flux field concentrators were installed outside the copper tube. High-frequency induction heating equipment was used, with a heating frequency of 40 kHz and a current of 3000 A. The steel section moved at a speed of 2 m/min, and the heating time was 2 s. After the pre-formed section’s first pass, it first passed through the induction heating coil. At this time, using the principle of electromagnetic induction heating, 850 °C was selected to heat the corner of the section so that the temperature of the corner increased and the plasticity of the corner decreased with the increase in temperature. The section continued to pass through the second, third, and fourth passes to obtain the sharp-corner section. The on-site picture of the experiment is shown in Figure 9. An infrared thermal imager, model FOTRIC 858X, produced by Shanghai Thermal Imaging Technology Co., Ltd. (Shanghai, China), was used to measure the heating temperature of the outer surface of the sharp-edged steel. The thermal imaging picture is shown in Figure 10. The technical parameters of the infrared camera were as follows: thermal sensitivity, 50 mk (0.05 °C); temperature range, −20–1700 °C; measurement accuracy, ±2 °C; frame rate, 60 Hz; measured parameter emissivity, 0.9; reflected temperature, 20 °C; atmospheric temperature, 20 °C; relative humidity, 50%; target distance, 1.0 m; external optical temperature, 20 °C; and external optical transmittance, 1.0.

**Figure 11:**
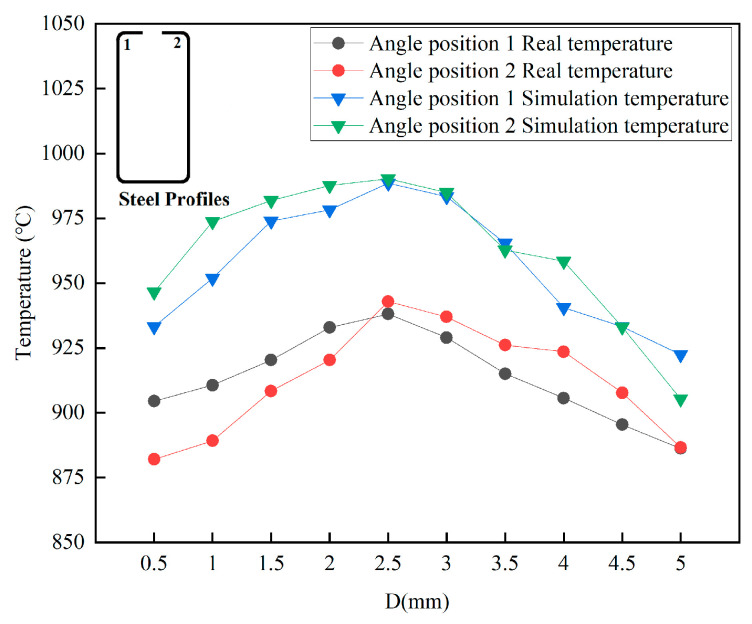
Temperature measurement results from infrared thermal imager and numerical simulation results.

Due to the symmetry of the coil, we only considered the infrared thermal imager temperature measurement results and numerical simulation results for corners 1 and 2 of the sharp-edged steel. The heating temperature obtained from the numerical simulation at corners 1 and 2 of the steel section was higher than the actual measurement results, as shown in Figure 11. As the actual measurement values are affected by the heat loss at the outer corner of the steel section, the thermal properties of the material play a crucial role in temperature variations. A constant surface heat transfer coefficient was used in the model. However, in reality, heat dissipates, leading to actual temperatures being lower than those from numerical simulations.

When using a heating frequency of 40 kHz and a current of 3000 A, we found that the relative errors between the simulated and measured temperatures at the highest temperature data points for corners 1 and 2 of the sharp-edged steel were 5.37% and 5.02%, respectively. The results indicate that the thermal properties of the sharp-edged steel are more appropriate under high-temperature conditions. Moreover, the finite element model has a particular capability in terms of prediction and is consistent with the actual situation.

## 6. Conclusions

This study employed numerical models and high-frequency induction heating experiments to conduct a detailed investigation into the induction heating process of sharp-edged steel and validate the simulation model. Furthermore, we delved deeply into the key factors affecting temperature, leading to the following conclusions:

(1) An electromagnetic–thermal field coupling model was established to study sharp-edged steel’s cold and hot composite forming process. The effects of different heating process parameters on the simulation results were analyzed, and experimental research based on optimized parameters was conducted. It was found that the relative errors between the simulated and measured heating temperatures were 5.37% and 5.02%, respectively. The results indicate that the finite element model has excellent predictive capability and can be used to guide actual production and process optimization.
(2) Using magnetic flux field concentrators can significantly enhance the magnetic field intensity of the simulation results. Therefore, installing magnetic flux field concentrators in local induction heating technology can effectively improve the efficiency of local induction heating.
(3) The simulation results indicate that the peak magnetic induction intensity is proportional to the heating frequency. This process’s optimal heating frequency, current, and heating time are 40 kHz, 3000 A, and 2 s, respectively. It can heat the center temperature of the cross section of the sharp-edged steel corner to 850 °C, and the magnetic flux density B reaches 2.51 T.
(4) Based on the experimental results, using the heating process parameters optimized through the numerical simulation, the heating temperature matched the expected values, indicating that cold and hot composite forming technology could be adopted to produce sharp-edged steel profiles for the curtain wall industry. Moreover, the dimensional accuracy of the sharp-edged steel was also high, indicating that it meets the requirements for engineering applications.
(5) The mechanism of the influence of process parameters on the temperature, such as heating current, frequency, and distance between the induction coil and the steel profile, requires further research.

## Data Availability

Not applicable.
